# Preconditioning with Associated Blocking of Ca^2+^ Inflow Alleviates Hypoxia-Induced Damage to Pancreatic β-Cells

**DOI:** 10.1371/journal.pone.0067498

**Published:** 2013-07-25

**Authors:** Zuheng Ma, Noah Moruzzi, Sergiu-Bogdan Catrina, Ingrid Hals, José Oberholzer, Valdemar Grill, Anneli Björklund

**Affiliations:** 1 Department of Molecular Medicine and Surgery, Karolinska Institutet, Stockholm, Sweden; 2 Institute of Cancer Research and Molecular Medicine, The Medical Faculty, Norwegian University of Science and Technology, Trondheim, Norway; 3 Department of Transplant/Surgery, University of Illinois at Chicago, Chicago, Illinois, United States of America; 4 Department of Endocrinology, St. Olav University Hospital, 7006 Trondheim, Norway; Universidad Miguel Hernández de Elche, Spain

## Abstract

**Objective:**

Beta cells of pancreatic islets are susceptible to functional deficits and damage by hypoxia. Here we aimed to characterize such effects and to test for and pharmacological means to alleviate a negative impact of hypoxia.

**Methods and Design:**

Rat and human pancreatic islets were subjected to 5.5 h of hypoxia after which functional and viability parameters were measured subsequent to the hypoxic period and/or following a 22 h re-oxygenation period. Preconditioning with diazoxide or other agents was usually done during a 22 h period prior to hypoxia.

**Results:**

Insulin contents decreased by 23% after 5.5 h of hypoxia and by 61% after a re-oxygenation period. Preconditioning with diazoxide time-dependently alleviated these hypoxia effects in rat and human islets. Hypoxia reduced proinsulin biosynthesis (^3^H-leucine incorporation into proinsulin) by 35%. Preconditioning counteracted this decrease by 91%. Preconditioning reduced hypoxia-induced necrosis by 40%, attenuated lowering of proteins of mitochondrial complexes I–IV and enhanced stimulation of HIF-1-alpha and phosphorylated AMPK proteins. Preconditioning by diazoxide was abolished by co-exposure to tolbutamide or elevated potassium (i.e. conditions which increase Ca^2+^ inflow). Preconditioning with nifedipine, a calcium channel blocker, partly reproduced effects of diazoxide. Both diazoxide and nifedipine moderately reduced basal glucose oxidation whereas glucose-induced oxygen consumption (tested with diazoxide) was unaffected. Preconditioning with diaxoxide enhanced insulin contents in transplants of rat islets to non-diabetic rats and lowered hyperglycemia vs. non-preconditioned islets in streptozotocin-diabetic rats. Preconditioning of human islet transplants lowered hyperglycemia in streptozotocin-diabetic nude mice.

**Conclusions:**

1) Prior blocking of Ca^2+^ inflow associates with lesser hypoxia-induced damage, 2) preconditioning affects basal mitochondrial metabolism and accelerates activation of hypoxia-reactive and potentially protective factors, 3) results indicate that preconditioning by K^+^-ATP-channel openers has therapeutic potential for islet transplantations.

## Introduction

Pancreatic beta cells display a high degree of oxidative metabolism. This makes their function and survival very sensitive to hypoxia and even mild hypoxia is known to inhibit insulin release [1]. From a clinical perspective hypoxia in beta cells is of great concern, since it is a major factor behind the demise of beta cells following transplantation [2], [3], [4]. Elucidating the possibilities and requirements for hypoxia-adaptive processes in beta cells are bound to be important for on-going efforts to improve the so far limited success [5] of islet transplantation in diabetic patients.

The effects of hypoxia on beta cells and ways to minimize hypoxia-induced damage are only partly elucidated. Adaptation to - relative - hypoxia can be demonstrated in most tissues and constitutes a basic survival mechanism [6]. However, the conditions that are best suited for beta cell adaptation to hypoxia are poorly defined and the possibilities to enhance adaptation by pharmacological means are largely unexplored.

In the present study we first investigated the impact of a standardized exposure to hypoxia on beta cells. Parameters of function and survival were assessed before and after a period of re-oxygenation. We then tested for conditions prior to hypoxia (i.e. preconditioning) that could be protective against the negative impact of hypoxia. The K^+^-ATP-channel opener diazoxide has been shown to be protective in heart and nervous tissue [7]: it was therefore chosen for testing. Upon finding beneficial effects we proceeded to test for mechanisms as well as effects in transplantation settings.

## Materials and Methods

### Materials

Ultrapure sodium alginate, Pronova UP-LVG (>60% guluronic acid, viscosity 20–200 mPa·s, endotoxin <100 EU/g) was from Nova Matrix (Oslo, Norway). All other chemicals were from Sigma, St Louis MO.

### Methods

#### Ethics

All animal studies were approved by the Northern Stockholm Ethical Committee on Experimental Animal Care (236/08; 388/10; 335/11; 100/11; 88/11; 28/08) and performed in accordance with guidelines from the Swedish National Board for Laboratory Animals. The protocol used in Trondheim was approved by the Norwegian Animal Research Authority (Permit Number FOTS 3114). Human islets used in Stockholm were obtained from a Nordic network under conditions specified elsewhere [8]. Human islets used in Trondheim were isolated at the Division of Transplant, University of Illinois (Chicago), and shipped to Norway. The protocol used in Trondheim was approved by the regional ethical committee in Norway (2011/782).

#### Animals

Male Sprague-Dawley rats were from Scanbur (Sollentuna, Sweden) and male NMRI nu/nu mice were obtained from Taconic (Denmark). Animals were maintained in a 12-h (0600–1800 h) light/dark cycle with free access to water and a standard diet. The rats weighed 250–350 g and the mice 35.5–49.3 g (33 weeks old) at the time of experiments.

#### Isolation, culture, and incubation of rat pancreatic islets

**Figure 1:**
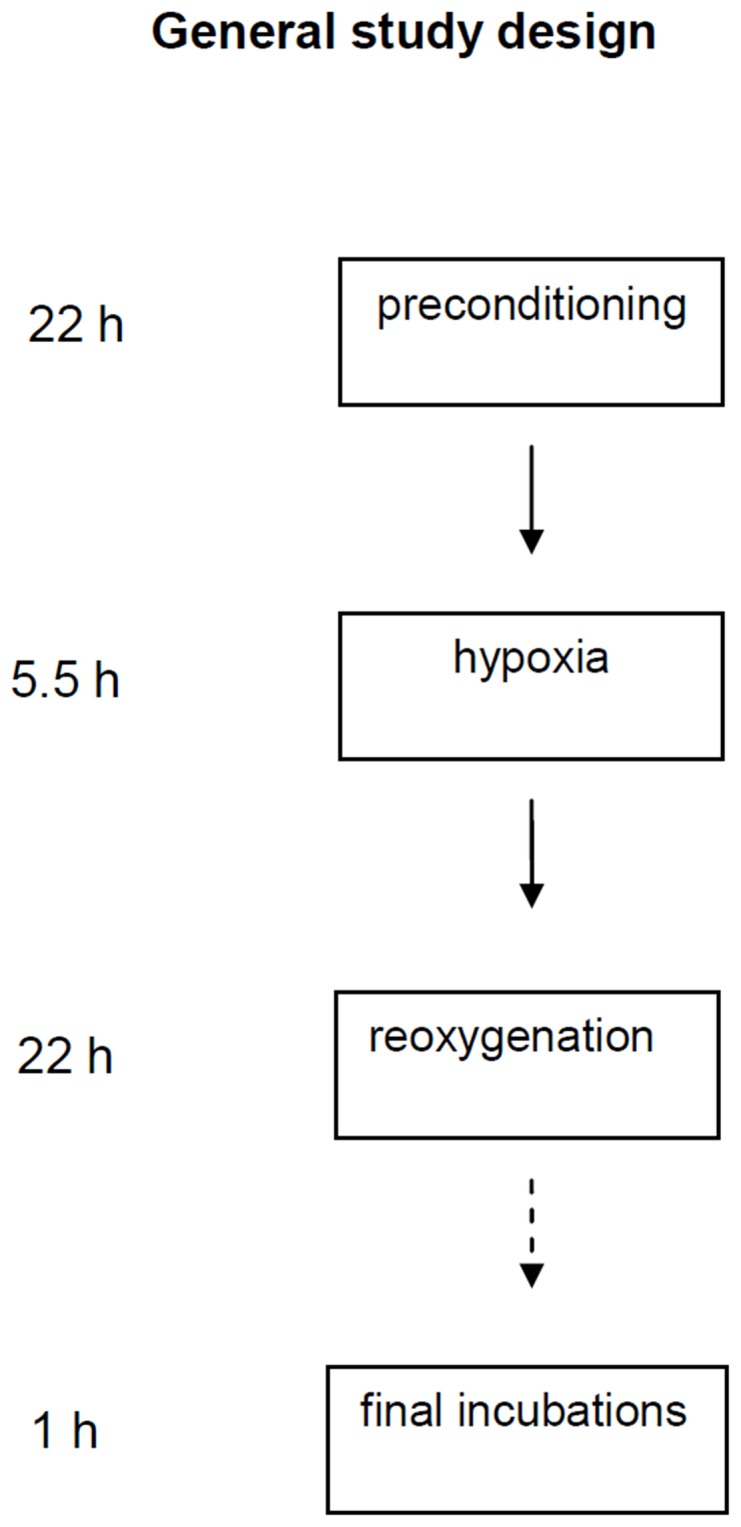
General study design. **Islets were cultured in 11 mmol/l glucose throughout with and without diazoxide and other additions (see text) for 22 h ( = preconditioning).** Islets were then exposed to hypoxia for 5.5 h ( = hypoxia). After hypoxia culture was continued in normoxia for 22 h ( = re-oxygenation). Constant normoxia was used as control. The re-oxygenation period was in most, but not in all, experiments followed by final incubations which included preincubation (30 min) followed by a glucose challenge (60 min).

Islets of Langerhans were isolated by collagenase digestion (Roche Diagnostics) followed by sedimentation. The general design of the experiments is depicted in Fig. 1. Islets were selected under a stereomicroscope. Care was taken to select only medium sized islets. Islets were transferred to 5 ml Petri dishes (Sterilin, Teddington, U.K.) containing RPMI-1640, 2 mmol/l glutamine, 10% (vol/vol) FCS, 100 units/ml benzylpenicillin, 0.1 mg/ml streptomycin, and 11 mmol/l glucose, each with or without the co-presence of 325 µmol/l diazoxide and/or other test substances as specified by each protocol. After this initial period of culture ( = preconditioning period) the medium was changed to RPMI with 11 mmol/l glucose. Islets were then cultured free-floating for 5.5 h, usually at 37°C, either during continued normoxia in an atmosphere of 5% CO_2_ in air or during hypoxia ( = hypoxia period). Hypoxia was achieved by a hypoxia chamber (Billup-Rothenberg, Calif). It was equilibrated with a gas mixture of 95% nitrogen and 5% CO_2_, achieving a stable reduction of oxygen to 0.8% during the standard experimental set-up. Islets, whether exposed to hypoxia or not, were then transferred to new dishes with the same composition of RPMI and cultured for another 22 h during normal (i.e. normoxic) conditions ( = re-oxygenation period).

#### Human islets incl. microencapsulation

Pancreata were obtained from normoglycemic cadaveric donors after appropriate consent for multiorgan donation. Islets were cultured for 48 h in 5.5 mmol/l glucose before experiments.

Microencapsulation was performed basically as described by Strand et al [9]. Capsule diameters (mean ± SEM, n = 33) were measured to 521±10 µmeter. After 4 days of culture, two suspensions of microencapsulated islets (3600 IEQ each) were incubated in culture media supplemented with either diazoxide (to a final concentration of 325 µmol/l) or an equal volume of 0.1 mol/l NaOH (control islets) for 48 h. Aliquots containing 600 IEQ each were collected for transplantation.

#### Transplantations

Three types of experiments were performed.

1. Non-diabetic rats: After culture for 22 h with or without 325 µmol/l diazoxide in vitro 50–150 islets were transplanted under the left kidney capsule as described [10]. Five days later the recipient rats were sacrificed and the transplants cut out and processed as described below.
2. Streptozotocin-diabetic rats: Equal weight male Sprague-Dawley rats were injected intraperitoneally (i.p.) with streptozotocin. Rats with blood glucose level consistently above 16 mmol/l were used as recipients. Islets from non-diabetic rats were cultured overnight with 11 mmol/l glucose with or without 325 µmol/l diazoxide or 10 µmol/l of nifedipine. Equal amount of islets (1200–1600 islets for each rat) were transplanted i.p. Levels of blood glucose were measured by AccuCheck Aviva (Roche Diagnostics).
3. Streptocotozin-diabetic nude mice: Diabetes was induced by i.p. injection of 150 mg/kg of streptozotocin. All mice that were used for experiments had blood glucose levels above 19 mmol/l 3 days after streptozotocin. For transplantations equal weight male NMRI nu/nu mice were anaesthetized with isofluran. A 0.3 cm abdominal incision was made to infuse encapsulated (48 h preculture ±325 µmol/l diazoxide) human islets (600 IEQ) i.p. Blood glucose and weight were measured every day during a 14 day period post transplantation.

#### Immunoreactive insulin

Immunoreactive insulin (IRI) was measured by RIA [11]. Cross-reactivity with human proinsulin was about 80%. Isolated islets and transplants were extracted for IRI in acid-ethanol [12].

#### Degradation of insulin

A pulse-chase method was employed, basically as described [13].

#### Proinsulin

Proinsulin was measured by the Rat/Mouse proinsulin ELISA kit (Mercordia, Uppsala, Sweden) according to the manufacturer's instructions. According to the manufacturer, this assay is specific for rat and mouse proinsulin I and II.

#### Western blotting

The procedure is described in [14]. For mitochondrial proteins we refrained from boiling the samples. Samples containing 30 (mitochondrial complexes I, II and IV) and 10 (complexes III and V, and beta actin) µg protein were prepared. Primary antibodies were used at the following dilutions: 1∶2,000 for HIF-1α (Novus Biologicals, Inc. USA), 1∶2,000 for AMPKα and phosphorylated AMPKα (Cell Signaling Technology, MA, USA), 1∶20,000 for beta-actin (Sigma) and 1∶500 for oxidative phosphorylation complexes 1–5 MS604 (MitoSciences, Eugene, OR, USA).

#### Protein biosynthesis

We measured the incorporation of [^3^H]-leucine into proinsulin and total islet proteins [15].

#### Glucose oxidation

Production of ^14^CO_2_ from D-[U-^14^C] glucose was measured basically as described [16].

#### Oxygen consumption rate (OCR)

This was measured employing Sea-Horse technology basically as described [17]. Seventy equally sized islets were used for each well.

#### ATP and ADP

An ATP Bioluminescence Assay Kit HS II (Roche) was used. ADP was measured after enzymatic elimination of ATP (to AMP) followed by conversion of ADP to ATP as described [18].

#### Total DNA, mitochondriaDNA/nuclearDNA and protein

Total DNA was measured by a fluorescent DNA Quantitation Kit (Bio-Rad) and protein content by the DC Protein Assay Kit (Bio-Rad).

For mitochondrial (mt)DNA islet DNA was extracted by DNeasy Blood and Tissue kit (Qiagen, Sweden). Mitochondrial relative DNA copy number was determined by calculation of the mtDNA/nuclear (n) DNA to the control ratio (11 mmol/l glucose without hypoxia) [19]. mtDNA-encoded NADH dehydrogenase 2 gene (ND-2) served as marker for total mtDNA and 18S rDNA for nDNA. Primers and real-time PCR kit were from Applied Biosystems.

#### Necrosis and apoptosis

The Cell Death Detection ELISA^PLUS^ kit (Roche Diagnostics) was used. Samples were placed into streptavidin-coated microplates and incubated with a mixture of anti-histone-biotin and anti-DNA-peroxidase. Islet culture media were analyzed for necrosis and islet lysates for apoptosis.

#### Statistical analysis

Results are expressed as mean ± SEM. Significant differences were tested using Student's paired t test (two-sided) or, for multiple comparisons, one way ANOVA. The Student-Newman-Keuls method was used for post-hoc analysis. A *P* value<0.05 was considered significant.

## Results

### Hypoxia induces lasting effects on IRI secretion and cellular contents

**Figure 2:**
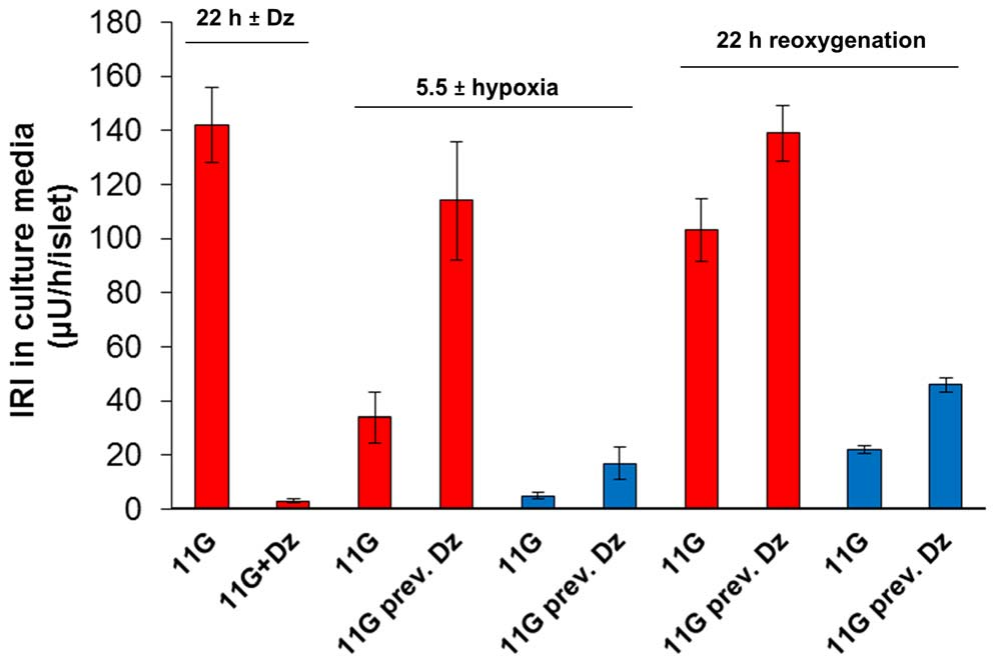
Effects of hypoxia on insulin accumulation in culture media. Shown are effects during preconditioning (22 h±diazoxide), 5.5 h of normoxia/hypoxia (± previous diazoxide) and subsequently 22 h of re-oxygenation (previous normoxia/hypoxia ± previous diazoxide). Mean ± SEM of four experiments. Red columns: normoxia;blue columns: hypoxia.

**Figure 3:**
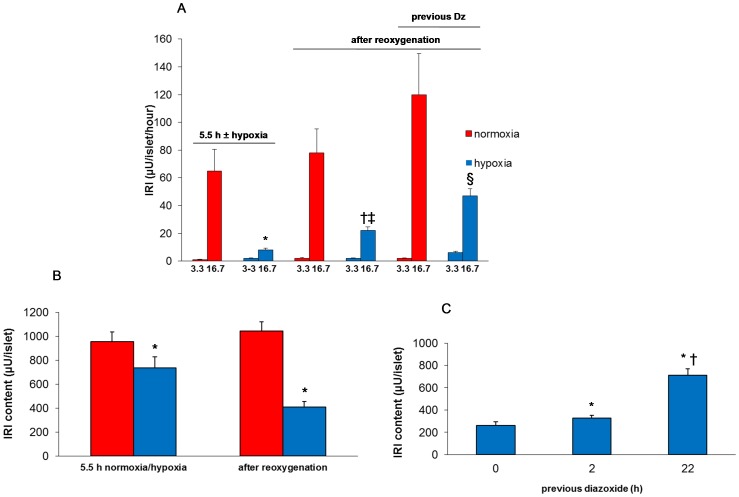
Effects of hypoxia and diazoxide on insulin secretion and islet insulin content. Shown are in **A** immediate effects (left part of figure) by 5.5 h of hypoxia on secretion and late effects, i.e. after re-oxygenation (right part of figure) including preconditioning with diazoxide (Dz). Glucose-induced insulin secretion was assessed in incubations with 3.3 and 16.7 mmol/l glucose. **B** and **C** depict islet insulin content. Mean ± SEM of seven experiments. In A: *P<0.02 vs. normoxia; ^†^ *P*<0.01 vs. no re-oxygenation; ^‡^ *P*<0.02 vs. normoxia after re-oxygenation. In B: *P<0.02 vs. uninterrupted normoxia. In A: ^§^ *P* = 0.027 vs. no previous diazoxide. In C: *P<0.001 for an effect of 22 h and ^†^ *P*<0.01 for an effect of 2 h of previous diazoxide. Red columns: normoxia; blue columns: hypoxia.

A 5.5 h period of hypoxia (design of experiments depicted in Fig. 1) almost abolished the accumulation of IRI into the culture medium (Fig. 2). The IRI response to 16.7 mmol/l glucose was markedly reduced when tested in sequence to the hypoxic event, (Fig. 3A). The response was partly normalized after re-oxygenation but still diminished (to 28% of the response of islets cultured during continuous normoxia, Fig. 3A). Islet contents of IRI were reduced after the period of hypoxia (Fig. 3B) and further reduced following the period of re-oxygenation (from 1044±77 to 409±44 µU/islet, Fig. 3B).

We tested the possibility of hypoxia accelerating the degradation of cellular insulin. Islets were labelled with [4,5-^3^H] leucine for 48 h and then pulse-chased. Duplicate measurements of insulin-antibody-precipitated radioactivity showed no decrease due to 5.5 h of hypoxia whether tested immediately after hypoxia or after the re-oxygenation period (results not shown).

During a lesser degree of hypoxia, i.e. exposure to 2.7–3.0% of oxygen the release of insulin into the culture medium was reduced by 83%. This inhibition was similar to that achieved by 0.8% of oxygen. Previous hypoxia slightly increased basal secretion in batch type incubations performed after re-oxygenation (p<0.04). Glucose-induced insulin secretion was however not altered (mean -5.3±4.3%). In contrast, insulin contents were clearly reduced by the lesser degree of hypoxia (from 840±173 to 573±114 µU/islet, *P* = 0.002, n = 4).

### Pre-exposure to diazoxide protects against hypoxia-induced reduction of insulin contents

The 22 h period of pre-exposure to diazoxide modestly improved a glucose-induced insulin response as measured after the re-oxygenation period (Fig. 3A). The effect by preconditioning on islet insulin contents was much more profound. Insulin contents were 2.7 fold increased relative to hypoxia-exposed islets, which had not been pre-treated for 22 h with diazoxide (Fig. 3C, compare left and right columns). The effect of diazoxide on insulin contents was not paralleled by diminished secretion during the re-oxygenation period (Fig. 2).

A 2 h pre-exposure to diazoxide exerted only a minor effect on islet insulin contents following re-oxygenation (Fig. 3C, middle column). No effect was seen when a 2 h exposure to diazoxide was followed by 22 h of normoxia before hypoxia (284 vs. 283 µU/islet without previous diazoxide, mean of two experiments). When diazoxide was present during the 5.5 h period of hypoxia - but not present before hypoxia - we found only a tendency for a minor increase in IRI insulin contents after re-oxygenation (increase +21±6%, *P* = 0.086, n = 4).

Pre-exposure to diazoxide did not affect glucose-induced insulin secretion when employing the lesser degree of hypoxia, i.e. exposure to 2.7–3.0% of oxygen However, diazoxide partly (by 59%) prevented the hypoxia-induced reduction in insulin contents, *P* = 0.01 vs. no previous diazoxide, n = 4.

### Effects on proinsulin

The 5.5 h period of hypoxia markedly reduced islet proinsulin contents (Fig. S1A). The decrease was similar in pre-conditioned and in non-preconditioned islets, 85±4% and 66±7% respectively. Re-oxygenation increased proinsulin contents by 168±58%, *P*<0.01 in pre-conditioned and non-significantly by 55±40% in non-preconditioned islets. Ratios of proinsulin to IRI after re-oxygenation were lower in pre-conditioned vs. non pre-conditioned islets (Fig. S1B).

### Reduced insulin biosynthesis is a sequel of hypoxia and is partially reversed by diazoxide

**Figure 4:**
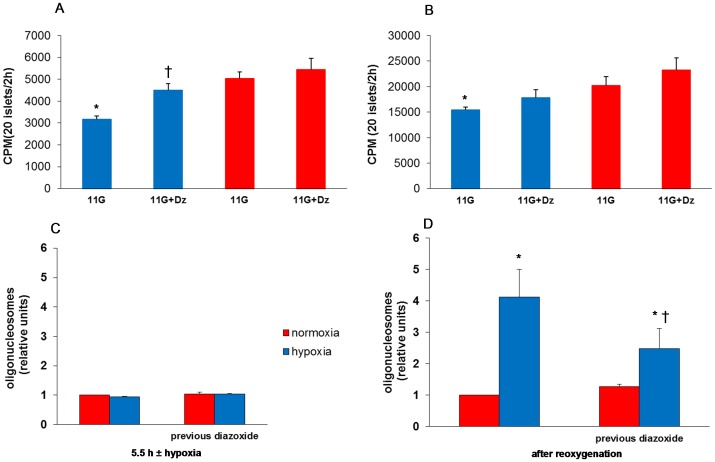
Effect of hypoxia and preconditioning with diazoxide (22 h) on biosynthesis and necrosis. **A** depicts insulin biosynthesis and **B**: total protein biosynthesis. Mean ± SEM of six experiments in A and B. In A: *P = 0.01 vs. normoxia, †*P*<0.03 vs. no previous diazoxide. In B: *P = 0.01 vs. normoxia. **C** depicts effect of preconditioning with diazoxide on necrosis measured in cell culture supernatants directly after hypoxia and **D** when measured after re-oxygenation. Mean ± SEM of four experiments in C and D. In D: *P<0.05 vs. normoxia, †*P*<0.05 vs. no previous diazoxide. Red columns: normoxia; blue columns: hypoxia.

Islets that had experienced hypoxia displayed diminished proinsulin biosynthesis (by 35±6%), after the re-oxygenation period (Fig. 4A). Total protein biosynthesis appeared to be depressed to a lesser extent (by 21±6%, Fig. 4B). Importantly, the decrease in proinsulin biosynthesis was partially and significantly reversed by preconditioning with diazoxide (Fig. 4A).

### Preconditioning reduces hypoxia-induced cell death

Necrosis was not observed when measured immediately after hypoxia but was apparent after re-oxygenation (Figs. 4C+D) Apoptosis was increased 2.3±0.4 fold, n = 4, *P*<0.05). Preconditioning reduced cell necrosis by 40%, (Fig. 4D) but did not affect an elevated rate of apoptosis (results not shown).

### Mitochondrial effects

**Figure 5:**
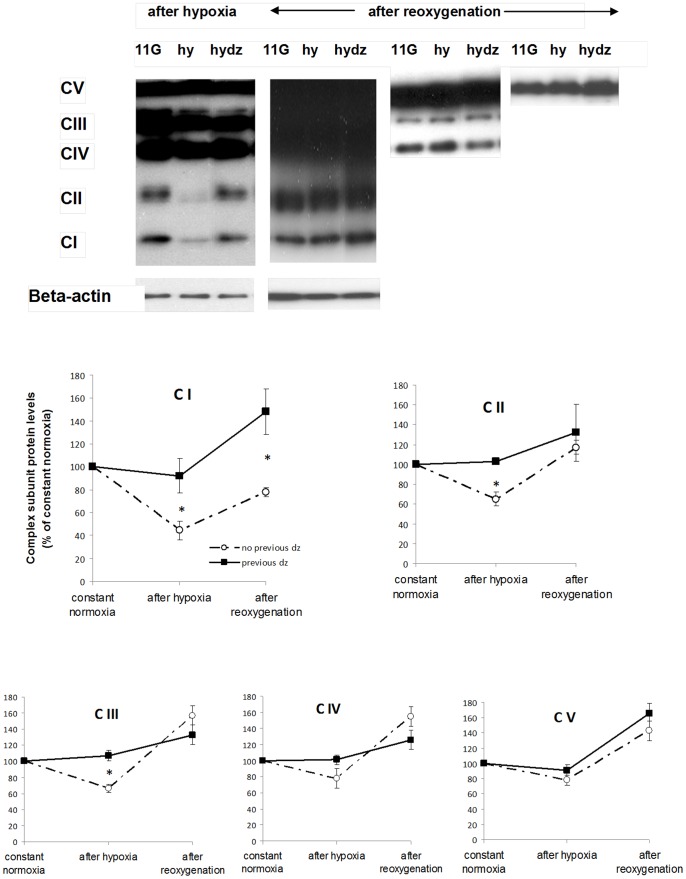
Effects of hypoxia on mitochondrial complexes I–V and modifying influences of preconditioning. Immunoblotting (for details see Methods) of sub-units of the complexes were performed on islets that were harvested after hypoxia or after 22 h of re-oxygenation. Typical Western blots from one experiment are shown in the upper part of the figure. Line charts show mean ± SEM of five (after hypoxia) or six (after re-oxygenation) individual experiments and are expressed as percentage of results in islets constantly cultured at normoxia. *P<0.05 or less vs. no previous diazoxide. 11G = 11 mmol/l glucose; hy = hypoxia, hydz = hypoxia+diazoxide.

Effects on insulin biosynthesis could be related to perturbations in energy production. We therefore tested for hypoxia-induced mitochondrial dysfunctions and modulation by diazoxide. Hypoxia severely reduced proteins of mitochondrial complexes I–V (Fig. 5). After re-oxygenation complexes II, III, IV and V were instead up-regulated whereas a decrease in complex I persisted. The effects of hypoxia before and after re-oxygenation were, in general, dampened by previous diazoxide. Importantly, preconditioning with diazoxide lessened the decrease of complex I after hypoxia as well as after re-oxygenation (Fig. 5).

**Figure 6:**
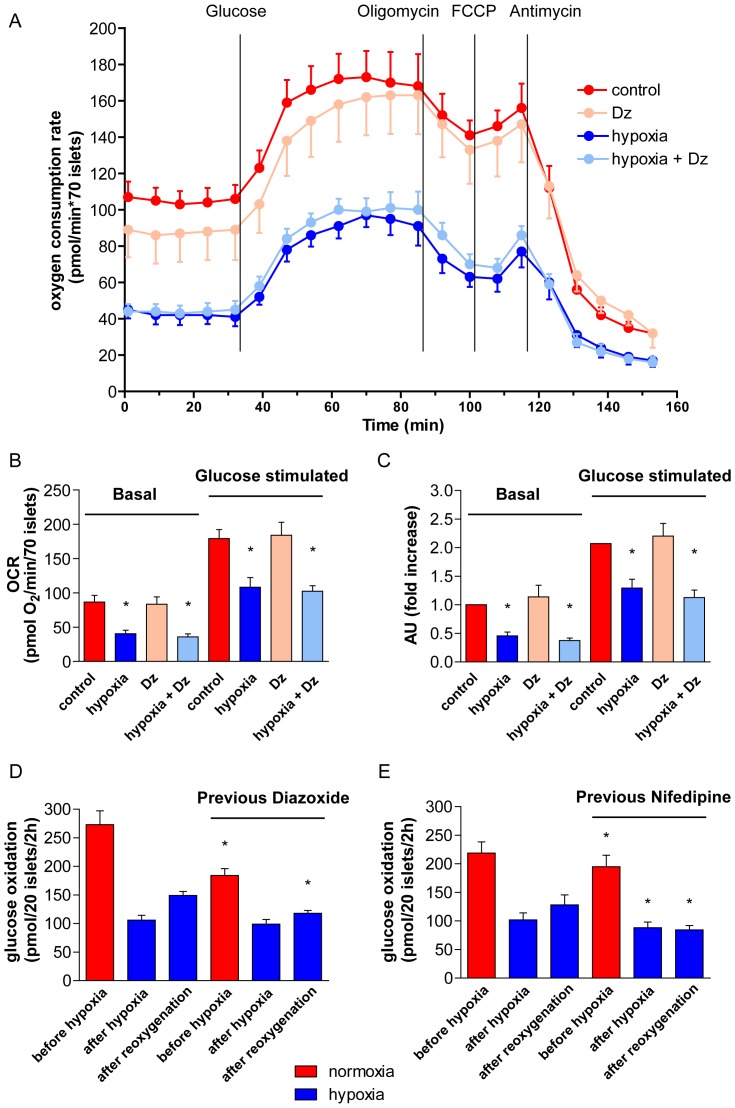
Oxygen consumption rates (A–C) and glucose oxidation (D and E). **Oxygen consumption was measured after the re-oxygenation period by a “Sea Horse” instrument (A–C).** In **A**, mean and SEM are shown from islets of four rats which were - separately for each rat - placed in parallel wells and processed in the same experiment. B presents a summary of basal and glucose-stimulated oxygen consumption from ten experiments, measured at basal (3.3 mmol/l) and glucose stimulated (16.7 mmol/l) respiration. In **C**, values from (**B**) were normalized to basal O_2_ consumption (11 mmol/l glucose). *P<0.05 or less vs. no previous hypoxia. D and E depict effects on glucose oxidation by preconditioning with diazoxide (**D**) or nifedipine (**E**). Glucose oxidation was measured at 11 mmol/l glucose closely before and after hypoxia as well as after re-oxygenation. Mean ± SEM of six experiments in D and E: *P<0.05 for effect of previous diazoxide or nifedipine.

Previous hypoxia decreased basal oxygen consumption (by 53±6%, n = 10, Fig. 6 A, B, C). The relative increase in response to glucose was however not negatively affected. Preconditioning with diazoxide did not alter the effects by hypoxia on oxygen consumption in response to glucose. Addition of oligomycin and FCCP qualitatively produced expected effects both with and without previous hypoxia, however the inhibitory effect of oligomycin did not level off even after 45–60 min exposures (data not shown), thus precluding calculations of coupled vs. uncoupled respiration. The ratio mtDNA/nDNA was decreased by hypoxia and was also slightly decreased by diazoxide both before and after hypoxia (Table 1). Levels of ATP and ADP were decreased even when taking into account a hypoxia-induced decrease of total DNA (Table 1). Preconditioning did not affect these parameters.

**Table 1:** ATP, ADP, protein, total DNA and mitochondrial DNA/nuclear DNA in islets immediately after hypoxia and after re-oxygenation.

	ATP (pmol/islet)	ADP (pmol/islet)	protein (µg/islet)	DNA (ng/islet)	ATP/DNA (nmol/mg DNA)	mDNA/nDNA (ratio)
		immediately after hypoxia				
normoxia	4.89±0.5	0.42±0.1	0.24±0.01	8.94±0.7	6.61±0.6	
normoxia+preconditioning	4.21±0.3	0.52±0.1	0.29±0.003	8.92±1.3	7.12±0.7	
hypoxia	1.20±0.02*	0.03±0.004*	0.20±0.01*	6.45±1.2*	3.20±0.1*	
hypoxia+preconditioning	1.31±0.02*	0.09±0.01*	0.24±0.01*	6.48±1.4*	4.15±0.1*	
		after re-oxygenation				
normoxia	4.9±0.1	0.25±0.02	0.27±0.01	9.67±1.3	6.85±0.1	1.0
normoxia+preconditioning	4.92±0.5	0.27±0.01	0.27±0.03	9.55±1.7	7.95±1.3	0.87±0.12
hypoxia	2.5±0.01*	0.11±0.02*	0.14±0.01*	5.54±1.0*	6.99±0.4	0.70±0.13*
hypoxia+preconditioning	2.01± 0.1*	0.10±0.01*	0.14±0.01*	5.86±1.1*	5.49±0.6	0.52±0.08*

* *P*<0.05 vs. no hypoxia, n = 6 except for protein (n = 3) and for mDNA/nDNA (n = 5).

Hypoxia markedly depressed glucose oxidation, both when measured closely in time after the exposure and after re-oxygenation (Fig. 6D). Regulation by glucose was preserved (data not shown). Preconditioning with diazoxide was associated with a moderate reduction of oxidation, when measured before hypoxia (Fig. 6D). No effect vs. hypoxia alone was evident when measured sequentially after hypoxia. However, basal oxidation was more depressed when measured after re-oxygenation than after hypoxia alone. On the other hand the glucose-induced increase in oxidation was unaltered (data not shown).

Considering our results on glucose oxidation we tested the possibility that a reduction of ATP synthesis prior to hypoxia could exert a protective effect. However, a 22 h preconditioning period with 2 nmol/l oligomycin, which inhibits ATP synthesis, did not induce preconditioning (insulin content being 428±61 vs. 285±27 µU/islet with oligomycin present during the preconditioning period, n = 4). (For optimizing the oligomycin concentration we performed dose-response experiments testing 2, 20 and 200 nmol/l in culture of rat islets together with 11 mmol/l glucose over night. Glucose induced insulin secretion in batch incubations the following day showed that secretion was inhibited by approximately 50% already at the lowest concentration of oligomycin and that higher concentrations resulted in almost complete inhibition and elevated basal levels indicating generalized toxicity).

### The preconditioning effect does not depend on de novo protein synthesis

The requirement for long term exposure to diazoxide for hypoxia-protective effects could indicate dependence on protein synthesis. However, co-exposure to the protein synthesis inhibitor cycloheximide (10 µmol/l) during 22 h of preconditioning did not affect preconditioning (data not shown).

### The preconditioning effect of diazoxide is abolished by co-culture with tolbutamide and elevated potassium and partly mimicked by nifedipine

**Figure 7:**
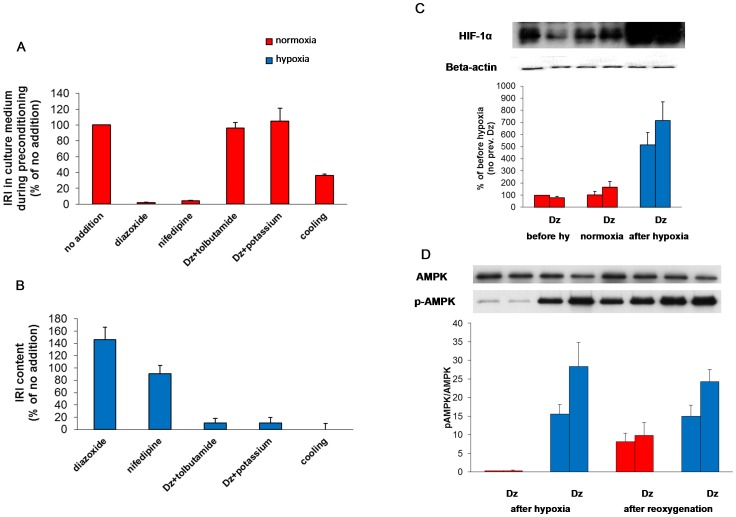
Influence on preconditioning by conditions affecting inflow of Ca^2+^ and/or insulin secretion (A–B), HIF1α (C) and AMPK (D). In A and B preconditioning was performed with diazoxide (dz) in the presence or absence of tolbutamide (100 µmol/l) or potassium (30 mmol/l). Also tested were effects of nifedipine (10 µmol/l) and cooling (28°C). **A** depicts insulin secreted to the culture medium expressed as % of no addition. **B** depicts islet insulin contents measured after 5.5 h of hypoxia followed by re-oxygenation. Incremental effects are shown as % of hypoxia only. Absolute values for Fig. 7A and B are shown in Text S1. Mean ± SEM of four experiments. **C** depicts effects of preconditioning with diazoxide (dz) on HIF1α protein (Western blot) before hypoxia and after 5.5 h of normoxia/hypoxia. Mean ± SEM of nine experiments, *P<0.05 vs. no previous diazoxide. **D** depicts effects of preconditioning with diazoxide (dz) by Western blotting on the ratio of AMPKα to phosphorylated AMPKα (p-AMPKα) in sequence to hypoxia and after re-oxygenation. Mean ± SEM of three experiments, *P<0.05 vs. no previous diazoxide. Representative blots are shown. Red columns: normoxia; blue columns: hypoxia.

Tolbutamide closes K^+^-ATP dependent channels, thus exerting the opposite effect of diazoxide on these channels. It was therefore of interest to determine whether co-culture with tolbutamide influenced preconditioning. The concentration of tolbutamide (100 µmol/l) was chosen to abolish the inhibitory effect by diazoxide on glucose-induced insulin secretion during the preconditioning period (mean of n = 4, Fig. 7A). Tolbutamide totally abolished preconditioning by diazoxide on cellular insulin contents (Fig. 7B).

Tolbutamide could exert its effects by a primary action on K^+^-ATP-channels or by a secondary effect on Ca^2+^ inflow through voltage-dependent calcium channels. These alternatives were tested by adding 30 mmol/l potassium during the preconditioning period. Elevated concentrations of potassium would per se depolarize the cell membrane, thereby short-circuiting any influence of K^+^- ATP channels. Elevated potassium reversed the inhibitory effect of diazoxide during the preconditioning period (n = 4, Fig. 7A) and reversed the preconditioning effect on islet insulin content (Fig. 7B).

To further document an association between inhibition of Ca^2+^ inflow and preconditioning we tested for effects by nifedipine which blocks Ca^2+^ inflow through L-type calcium channels. Preconditioning for 22 h with nifedipine (10 µmol/l) inhibited glucose-induced accumulation of insulin into the culture medium to a degree similar to that of diazoxide (Fig. 7A). Preconditioning with nifedipine protected against the hypoxia-induced decrease in islet IRI contents (Fig. 7B), again an effect similar to that of diazoxide (but somewhat less marked). Glucose-induced insulin secretion was only marginally improved (results not shown). Further, in analogy with diazoxide, nifedipine inhibited glucose oxidation to a moderate extent (Fig. 6E).

### Cooling-induced inhibition of exocytosis fails to exert protective effects

Cooling inhibits exocytosis much more severely than Ca^2+^inflow [20], [21], [22]. We employed cooling to separate effects on Ca^2+^ inflow from attendant effects on insulin secretion. The effects of cooling to 28 degrees were tested during the standard preconditioning period of 22 h at the standard glucose concentration of 11 mmol/l. Cooling markedly inhibited insulin secretion (by 64±2%) under these conditions (Fig. 7A). Such inhibition did not however, protect against the negative effects of hypoxia on insulin contents (Fig. 7B) or on glucose-induced insulin secretion (at 37 degrees, results not shown).

### Diazoxide enhances the induction of hypoxia inducible factor 1 alpha (HIF- 1 alpha) and AMPK

HIF-1-alpha and AMPK are recognized as universal factors in the adaptation to hypoxia (reviews [6], [23]). The levels of HIF-1-alpha protein were dramatically increased immediately after hypoxia from a baseline of a small but detectable signal during normoxia (Fig. 7C). Effects of diazoxide on HIF-1 alpha protein were complex. During normoxia the presence of the drug tended to decrease the HIF-1 alpha signal. The signal was however actually enhanced following 5.5 h of withdrawal of the drug (i.e. during continued normoxia). Immediately after 5.5 h of hypoxia the HIF-1 alpha protein was, as mentioned, markedly increased, but this increase was further enhanced by preconditioning with diazoxide (Fig. 7C).

Hypoxia markedly activated the phosphorylated form of AMPK, the ratio of phosphorylated to non-phosphorylated form increasing both immediately after hypoxia and after re-oxygenation (Fig. 7D). Preconditioning with diazoxide further increased this ratio directly after hypoxia as well as after re-oxygenation (Fig. 7D).

### A preconditioning effect of diazoxide can be reproduced in human islets in vitro

Limited access to human islets allowed us to test in a pilot fashion whether effects observed in rat islets could be reproduced in human islets. The protocol that we used was identical to the standard one for rat islets. In both of two preparations of human islets preconditioning with diazoxide increased the mean insulin content after hypoxia and re-oxygenation. The mean insulin content was 746 µU per islet in preconditioned islets as compared to 447 µU in islets that were not preconditioned.

### Preconditioning by diazoxide in vitro enhances IRI contents in islet transplants and lowers diabetes-induced hyperglycemia

**Figure 8:**
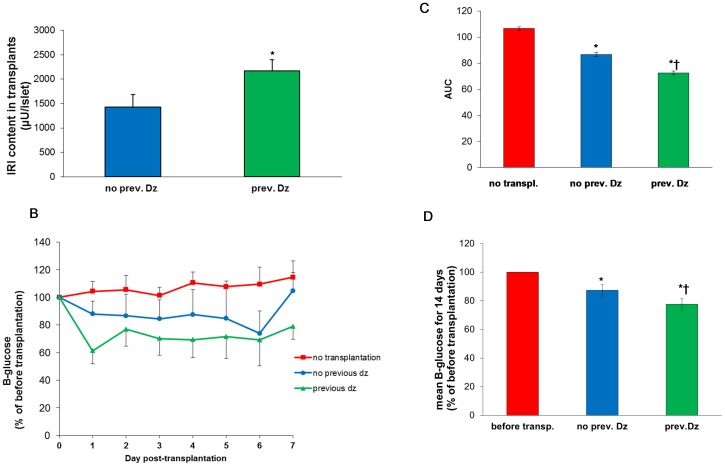
Effects in vivo by preconditioning in vitro. **A** shows effect of preconditioning with diazoxide on insulin content of islets (µU/islet) transplanted under the kidney capsule to non-diabetic rats. Transplants were taken out after five days. *P<0.05 vs. no previous diazoxide, mean ± SEM of six experiments. **B**: Effect of preconditioning with diazoxide on B-glucose levels in streptozotocin-diabetic rats transplanted i.p. with 1200–1600 islets. Mean ± SEM of four experiments. In **C** the AUC (area under the curve) calculated from **B** is shown. *P<0.05 vs. no transplantation and †*P*<0.05 vs. no previous diazoxide. Red squares: no transplantation; blue circles: transplantation but no previous diazoxide; green triangles: transplantation with islets pre-treated with diazoxide. **D**: Effect of preconditioning with diazoxide on B-glucose levels for 14 days (mean ± SEM) in streptozotocin-diabetic NMRI nu/nu mice transplanted i.p. with 600 encapsulated human islet equivalents. Mean ± SEM of five experiments. *P<0.05 vs. before transplantation and †*P*<0.05 vs. no previous diazoxide. Red columns: before transplantation; blue columns: transplantation but no previous diazoxide; green columns: transplantation with islets pre-treated with diazoxide.

We tested, as proof of concept, for relevance of preconditioning in vitro for the outcome of islet transplantation. The concept was tested in three different ways. In one transplantations were performed to non-diabetic rats. Islets from rats were cultured for 22 h with or without diazoxide in vitro and then transplanted under the kidney capsule of syngeneic non-diabetic rats. Five days after transplantation the grafts were cut out and insulin contents measured. The insulin contents were increased by 183±37% due to preconditioning (Fig. 8A).

The ability of preconditioning to affect the level of hyperglycemia in diabetes was tested in two models of streptozotocin diabetes. In the first model of diabetes we used streptozotocin diabetic rats. Injections of 1200–1600 rat islets i.p. without preconditioning were marginally effective in lowering blood glucose over a 7 day period (Fig. 8B+C). Preconditioned islets were significantly more effective in this respect. In the second model of diabetes we tested for preconditioning effects in human islets, which were transplanted i.p. to streptozotocin-diabetic nude mice. (Here we used alginate-encapsulated islets, since encapsulation increases the effectiveness of i.p. transplants, unpublished observations). There was also in this experimental model a stronger effect on hyperglycemia over a 14 day period in islets which had been subjected to preconditioning (Fig. 8D).

## Discussion and Conclusions

We demonstrate for the first time a robust preconditioning effect of diazoxide that partially protects against the negative impact by hypoxia on viability and insulin biosynthesis. We find that the beneficial effect is time dependent, associated with reduced Ca^2+^ inflow and reduction of glucose oxidation and a corrective influence on proteins of oxidative phosphorylation. We also provide proof of concept for a beneficial influence by preconditioning on the function of transplanted islets. Further, we extend previous observations on the general impact of hypoxia on beta cell function and survival.

We first wish to discuss the impact of hypoxia in general. We find, as others [1], that insulin secretion is blocked during hypoxia. Novel findings are that cellular insulin contents and biosynthesis are afflicted to a larger extent than responses to glucose in terms of insulin release, oxidation and O_2_ consumption. Negative effects of hypoxia on insulin biosynthesis were suggested by the hypoxia-induced marked decrease in insulin contents, by the profound decrease in islet proinsulin which was registered immediately after the hypoxic event, by direct measurements of ^3^H- leucine incorporation into proinsulin following re-oxygenation and, lastly, by the lack of enhancement of insulin degradation. Novel findings are also the acute and lingering impact by hypoxia on proteins of mitochondrial complexes as well as lowering of the mtDNA/nDNA ratio, the latter being in line with functional and/or structural reductions of beta cell mitochondria. One may speculate that such reductions could be more crucial for upholding biosynthesis than for secretory processes.

We next discuss the effects termed here preconditioning. A question to be answered before further discussion is what preconditioning stands for and whether agents and in particular diazoxide are retained after the pre-culture period, in which case one would observe a combined effect of the previous and a still present exposure to the agent. Our unpublished observations do not indicate a lingering presence of diazoxide, since the inhibitory effects on insulin secretion of a previous 22 h exposure to diazoxide are reversed within one hour. However, effects of putatively retained diazoxide which are not related to effects of K^+^ ATP channels cannot be completely excluded. It is with this reservation that we are using the term preconditioning throughout the study.

We note that the beneficial effects of previous diazoxide encompassed both function (insulin biosynthesis) and viability (necrosis). However, the concomitantly registered increase in apoptosis demonstrates that preconditioning with diazoxide was not able to wholly counteract on-going hypoxia-induced cellular damage.

As to mechanisms of preconditioning our results give evidence that preconditioning depends, at least in part, on inhibition of stimulated Ca^2+^ inflow. Thus, preconditioning was totally abolished not only when the diazoxide effect on K^+^-ATP channels was opposed by a sulphonylurea drug, but also by the co-presence of elevated potassium which induces inflow of Ca^2+^ without involving K^+^-ATP channels. Furthermore, nifedipine, which blocks Ca^2+^ inflow by a direct effect on L-type voltage-dependent calcium channels, reproduced to a large extent the beneficial effect of diazoxide on cellular insulin contents. Both diazoxide [24] and nifedipine [25] are known to lower cytosolic calcium in islets in the present dose and time (22 h) perspective. (Perhaps due to the somewhat weaker effects of nifedipine seen in vitro we were not able to obtain a significant beneficial effect in a transplantation protocol, results not shown). In this context it is interesting to note that another calcium blocker, verapamil, when administered in vivo reduced beta cell apoptosis and enhanced endogenous insulin levels [26].

The cooling experiments were designed to dissociate, at least partly, the effect of inhibition of Ca^2+^ inflow from inhibition of exocytosis. Lowering temperature into the range that we employed is bound to affect exocytosis to a much greater extent than Ca^2+^ inflow [20], [21], [22]. The 64% inhibition of insulin secretion by cooling that we observe did not protect against the hypoxia-induced lowering of insulin contents. This finding we interpret to rule out a major protective influence by inhibition of insulin secretion per se. In this context it is notable that a protective effect of an analogue of diazoxide against streptozotocin toxicity also occurred independently of effects on insulin secretion [27].

A toxic effect of a sustained elevation of cytosolic calcium is well established in other tissues and may be mediated by several mechanisms, some of which include the production of ROS [28]. In this context it is interesting that hypoxia in beta cells acutely increases intracellular calcium [29].

Notwithstanding the evidence for a role of calcium behind the preconditioning effects one should not dismiss the possibility of additional factors of importance. A direct effect of diazoxide on mitochondria (not tied to putative K^+^ ATP channels in mitochondria) has been demonstrated [30] and may be tied to the modest but significant lowering effects on mitochondrial metabolism that we observe. (The lack of correspondence between effects by diazoxide on oxidation and on ATP and ADP levels could possibly be explained by a decrease in energy expenditure). Reduced metabolism could in turn be related to enhancement of HIF-1 alpha and phosphorylated AMPK, which are recognized to be the two major hypoxia-sensing factors of cells [6]. As described in Results the effects of diazoxide on the HIF-1 alpha protein were complex, however the end results was an enhancing effect after hypoxia. It seems possible that an accelerated rise in HIF-1 alpha during hypoxia could lessen damage afflicted by hypoxia. Such notion is in line with the observation that forced over-expression of HIF-1 alpha improved the results of beta cell transplantation [31], [32]. As to effects on AMPK this parameter which is sensitive to changes in metabolism was enhanced by diazoxide in response to hypoxia, i.e. the ratio of phosphorylated to non-phosphorylated protein increased beyond the effects of hypoxia per se. In this context it is interesting to note that AMPK activation has been linked to protective effects of ischemic preconditioning [33] and could be another factor of importance for preconditioning by diazoxide.

Are the present results clinically relevant? The robustness of the diazoxide effect, and the beneficial effects of preconditioning seen post transplantation for both rat and human islets are all supportive. One may question the likelihood for merely a short term treatment in vitro to improve transplantation results long term.? However, the early time period after transplantation is known to determine the fate of transplanted islets to a large part. Several negative factors are then transiently operative and combine to eliminate the majority of islets [34]. Reducing the impact of one of these negative factors, i.e. hypoxia, could help more beta cells survive the initial and critical period after transplantation thereby increasing the potential for better transplant functioning in the long term. In any case, our data should give impetus for further studies in which the usefulness of preconditioning in vitro for the outcome of clinical transplantations in vivo can be extensively tested.

## Supporting Information

Figure S1 Effect of preconditioning with diazoxide measured after reoxygenation on **A**: islet proinsulin content. *P<0.05 vs. normoxia, †*P*<0.05 vs. before re-oxygenation. **B**: proinsulin expressed as % of total IRI. *P<0.05 vs. no preconditioning with diazoxide. Mean ± SEM of five experiments.
(TIF)

Text S1 **Supporting information to** **Fig. 7A** **and** **Fig. 7B** **.**
(DOC)
