# Supplementary figures and images for: Preconditioning with Associated Blocking of Ca2+ Inflow Alleviates Hypoxia-Induced Damage to Pancreatic β-Cells
Source: PLoS One. 2013 Jul 25;8(7):e67498. doi: 10.1371/journal.pone.0067498 (PMC3723782; doi:10.1371/journal.pone.0067498)

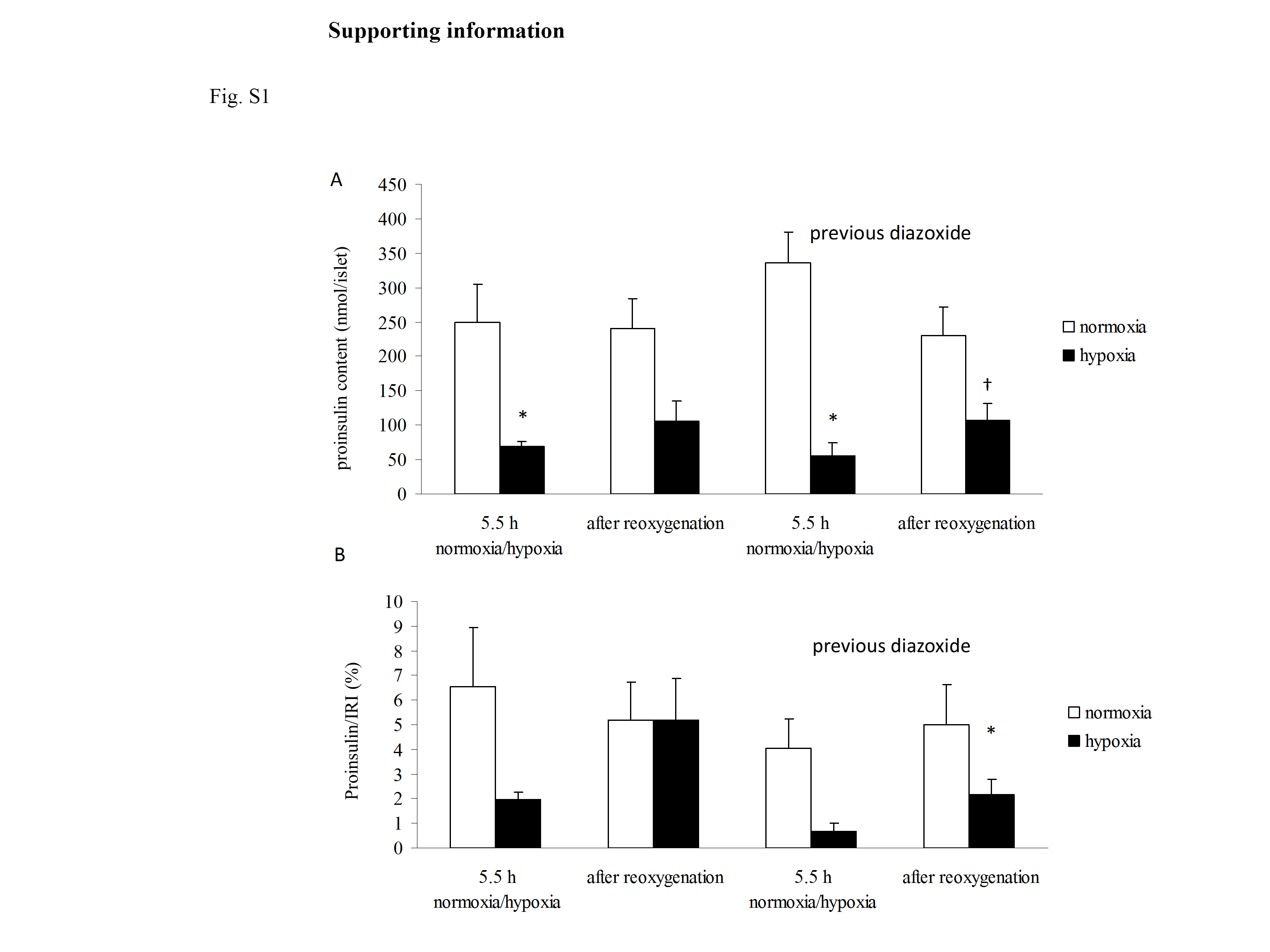

Supplement: Figure S1 — Effect of preconditioning with diazoxide measured after reoxygenation on A: islet proinsulin content. *P<0.05 vs. normoxia, †P<0.05 vs. before re-oxygenation. B: proinsulin expressed as % of total IRI. *P<0.05 vs. no preconditioning with diazoxide. Mean ± SEM of five experiments. (TIF) [file pone.0067498.s001.tif]
