# Supplementary material for: Preconditioning with Associated Blocking of Ca2+ Inflow Alleviates Hypoxia-Induced Damage to Pancreatic β-Cells
Source: PLoS One. 2013 Jul 25;8(7):e67498. doi: 10.1371/journal.pone.0067498 (PMC3723782; doi:10.1371/journal.pone.0067498)
Supplement: Text S1 — Supporting information to Fig. 7A and Fig. 7B . (DOC) [file pone.0067498.s002.doc]

**Supporting information**

**Supporting information to Fig. 7A**: Absolute values for inhibition of secretion were from 142 ± 14 to 3 ± 0.7 µU/islet/h (by diazoxide), from 101 ± 12 to 3 ± 0.4 (by nifedipine), from 115 ± 13 to 119 ± 3 (by diazoxide + tolbutamide), from 57 ± 3 to 59 ± 6 (by diazoxide + potassium) and from 100 ± 5 to 35 ± 1 µU/islet/h (by cooling).

**Supporting information to Fig. 7B**: Absolute values for increase in insulin content were from 388 ± 58 to 926 ± 20 µU/islet/h( diazoxide), 404 ± 36 to 775 ± 90 (nifedipine), 200 ± 28 to 205 ± 21 (diazoxide + tolbutamide), 314 ± 27 to 345 ± 34 (diazoxide + potassium) and 446 ± 17 to 441 ± 31 µU/islet (cooling).

**Fig. S1**: Effect of preconditioning with diazoxide measured after reoxygenation on **A:** islet proinsulin content. **P* <0.05 vs. normoxia, †*P* < 0.05 vs. before re-oxygenation. **B:** proinsulin expressed as % of total IRI. **P* <0.05 vs. no preconditioning with diazoxide. Mean ± SEM of five experiments
